# Hyperglycemia-stimulating diet induces liver steatosis in sheep

**DOI:** 10.1038/s41598-020-68909-z

**Published:** 2020-07-22

**Authors:** Mugagga Kalyesubula, Ramgopal Mopuri, Alexander Rosov, Tamir Alon, Nir Edery, Uzi Moallem, Hay Dvir

**Affiliations:** 1 Institute of Animal Science, Volcani Center - ARO, Rishon LeZion, Israel; 2 Department of Animal Science, The Hebrew University of Jerusalem, Rehovot, Israel; 3 Pathology Laboratory, Kimron Veterinary Institute, Veterinary Services, Rishon LeZion, Israel

**Keywords:** Non-alcoholic fatty liver disease, Fat metabolism, Metabolic diseases, Metabolic syndrome, Obesity

## Abstract

Hepatic steatosis is strongly associated with chronic liver disease and systemic metabolic disorder. Adipose lipolysis is a recognized principal source of intrahepatic fat in various metabolic disorders, including non-alcoholic fatty liver disease. We hypothesized that, in the premorbid state, hepatic de novo lipogenesis (DNL) driven by excess carbohydrates abundance might play a more significant role. We employed a novel nutritional model in sheep of two distinct carbohydrates abundances. During 4 months of the dietary treatment, lambs were monitored for metabolic and terminal liver parameters. Lambs grown on the high-calorie (HC) diet were consistently more hyperglycemic and hyperinsulinemic than lambs grown on the lower-calorie (LC) diet (*P* < 0.0001). As a result, the HC lambs developed systemic- (HOMA-IR of 7.3 vs. 3.1; *P* < 0.0001), and adipose- (ADIPO-IR of 342.7 vs. 74.4; *P* < 0.0001) insulin resistance, significant adiposity (*P* < 0.0001), and higher plasma triglycerides (*P* < 0.05). Circulating leukocytes in the HC lambs had higher mRNA expression levels of the proinflammatory markers *CCL2* (*P* < 0.01) and *TNF-alpha* (*P* < 0.04), and *IL1B* trended higher (*P* < 0.1). Remarkably, lambs on the HC diet developed substantial liver steatosis (mean fat content of 8.1 vs. 5.3% in the LC group; *P* < 0.0001) with a higher histological steatosis score (2.1 vs. 0.4; *P* < 0.0002). Hepatic steatosis was most-strongly associated with blood glucose and insulin levels but negatively correlated with circulating fatty acids—indicating a more significant contribution from hepatic DNL than from adipose lipolysis. Sheep may prove an attractive large-animal model of fatty liver and metabolic comorbidities resulting from excess carbohydrate-based energy early in life.

## Introduction

As the body hub for energy metabolism, the liver plays a crucial role in processing dietary energy, converting it from one form to another, and distributing it to extrahepatic energy-expenditure or energy-storage tissues. To support short-term energy demands, the liver also stores energy, mainly carbohydrates-based energy in the form of glycogen. Although fat is generally stored in adipose tissue for long-term energy requirements, some low steady-state level of hepatic fat is considered normal and can be utilized and recycled efficiently for short-term energy needs^1^. Fatty liver (FL), however, represent an abnormal condition of ectopic retention of intrahepatic fat (hepatic steatosis; > 5.5% w/w), which is strongly associated with chronic liver disease (steatohepatitis, cirrhosis), with prominent metabolic disorders such as obesity, type-2 diabetes and metabolic syndrome, and with systemic metabolic derangements such as dyslipidemia, hyperglycemia and insulin resistance^2–5^.

FL develops when the rate of acquisition of fatty acids (FA) from the circulation and from hepatic de novo lipogenesis (DNL) exceeds their rate of disposal by oxidation and export out of the liver. The resulting excess of hepatic FA is utilized in triglycerides (TG) synthesis for storage as intrahepatic fat in hepatocytes lipid droplets^6^. Yet, why intrahepatic fat starts to accumulate remains considerably unclear, and the governing molecular mechanisms are a subject of extensive investigations^1^.

Notably, adipose lipolysis releasing non-esterified fatty acids (NEFA) to the circulation in response to starvation^7–9^, or as a result of adipose insulin resistance developed by overnutrition^10,11^ or by excessive alcohol consumption^12^, is a common FL determinant believed to serve as a major substrate source for ectopic hepatic TG. Indeed, although carbohydrates can fuel hepatic DNL and thereby increase the hepatic pool of TG, in the settings of non-alcoholic fatty liver disease (NAFLD), adipose-derived NEFA accounted for most (~ 60%) of the hepatic TG whereas DNL accounted for only ~ 25%^13^. The relative contributions to ectopic hepatic TG in healthy or premorbid states were less explored, although they may better reflect the initiation phase of non-alcoholic liver steatosis.

Ruminants are also susceptible to the development of FL during late pregnancy and early lactation; however, it occurs on a background of negative energy balance that triggers excessive adipose lipolysis^14^. Although similar physiology develops in women with pregnancy starvation ketoacidosis^15^, this presentation of FL due to lack of dietary energy is of limited utility to represent the nature of NAFLD initiation by excessive caloric intake. Whether ruminants can develop hepatic steatosis due to positive energy balance based on excess carbohydrate energy is currently unknown.

In previous studies with sheep^16^, we found that feeding growing lambs a carbohydrates-rich grain-concentrate ration induces substantial hyperglycemia, with ~ 40% higher blood glucose levels than in lambs raised on a hay-based ration. Based on this observation and on the fact that sheep, as humans, are willing to overeat, we hypothesized that feeding them with that hyperglycemia-stimulating diet for an extended period will induce hepatic steatosis. Here, we tested this hypothesis to investigate long-term metabolic and liver-related implications of such dietary hyperglycemia, as well as to assess the potential of using sheep as a large-animal model for FL research with relevance to NAFLD.

## Results

### The HC diet induced hyperglycemia, hyperinsulinemia and insulin resistance

**Figure 1:**
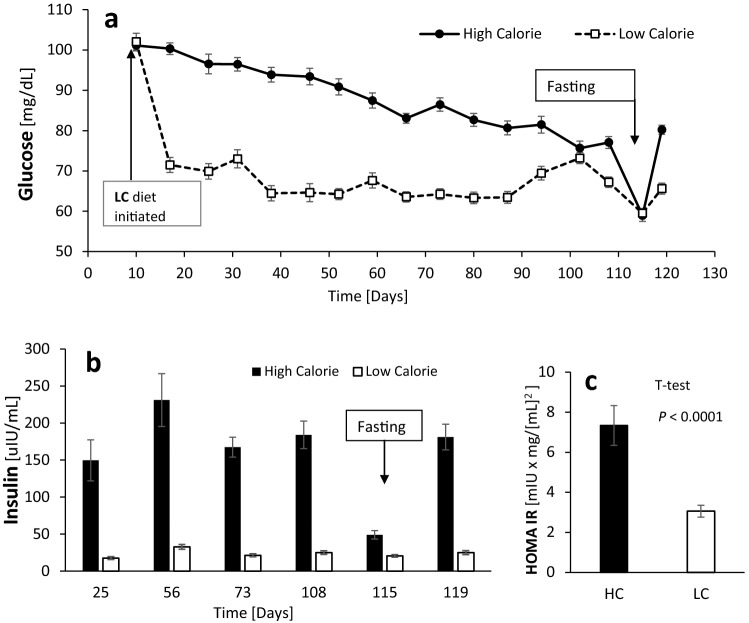
Glucose and Insulin Response to the dietary treatments. (**a**) Glucose response. Repeated measures ANOVA shows an effect of: treatment (*P* < 0.0001), time (*P* < 0.0001), treatment-time interaction (*P* < 0.0001) and individual animal (*P* < 0.0011). SEM = 0.638. The fasting glucose, at day 115 from treatment, was not included in the ANOVA. Both treatment groups were given a 10-day acclimatization period in the experimental pen with diets with similar metabolizable energy of ~ 4.86 MCal. (**b**) Insulin response. The analysis shows an effect of treatment (*P* < 0.0001), time (*P* < 0.0001), individual animal (*P* < 0.0101) but no treatment by time interaction. SEM = 8.29. Fasting insulin concentrations were higher in the HC group (*P* < 0.0002), but excluded from the overall ANOVA with repeated measures analysis. (**c**) Insulin resistance measured by HOMA-IR. Student’s t-test *P* < 0.0001; SEM = 0.6. Due to unequal variances between the treatment groups, the statistical analysis was performed on the log-transformed values for both (**b**) and (**c**).

Although both rations were carbohydrates-based diets with little fat content (under 3%) and served ad libitum, the HC diet yielded a significantly higher average intake of metabolizable energy (~ 6.3 vs. 3.5 Mcal/day, respectively; Table 1), thereby providing more glucose precursors for hepatic gluconeogenesis. Within a few days of treatment, the lambs on the HC diet developed substantially higher blood-glucose concentration than those on the LC diet (100 vs. 71 mg/dL, respectively ; *P* < 0.0001). The glycemic difference decreased mildly with time (Fig. 1a) but remained relatively high and steady throughout the 4-month experimental period, with overall mean fed-glucose concentrations of 86.2 vs. 68.6 mg/dL, respectively (*P* < 0.0001; Table 2). Noteworthy, postprandial blood glucose concentrations of ~ 100 mg/dL are considered normal in humans, yet they are exceptionally high in sheep having normal blood glucose concentrations of ~ 52 mg/dL^17^. Higher concentrations, typically above 60 mg/dL in our husbandry with the Afec-Assaf breed, are required to support lambs growth (Supplementary Figure 2).

**Table 1:** Composition of feeds expressed as dry matter.

Composition	Oat hay	Concentrate	Ground corn	Soybean meal
Moisture (%)	11.0	14.9	16.3	13.6
Crude protein (%)	10.0	18.4	9.4	53.0
Crude fat (%)	3	3.5	4.1	1.6
NDF (%)	56.0	22.6	9.2	8.0
ADF (%)	31.6	9.9	2.6	5.9
ME, MCal/kg	2.19	3.2	3.9	2.8
HC average intake (kg)	0.19 ± 0.01	1.81 ± 0.06	–	–
HC average intake (ME, MCal/kg)	0.41 ± 0.01	5.79 ± 0.18	–	–
LC average intake (kg)	0.98 ± 0.07	–	0.18 ± 0.01	0.22 ± 0.01
LC average intake (ME, MCal/kg)	2.11 ± 0.16	–	0.70 ± 0.04	0.62 ± 0.04

Crude protein intake was maintained at ~ 15% of the dietary intake for both the LC and HC rations. Values represent mean ± SD as relevant. *ADF* acid detergent fiber, *NDF* neutral detergent fiber, *ME* metabolizable energy.

**Table 2:** Effect of the high calorie and low-calorie treatments on body weight, glucose and insulin. Except for initial glucose, the values represent overall mean of the experimental period.

Parameter	Treatment		SEM	*P-*value		
	High calorie	Low calorie		Treatment	Time	Treatment × time
Initial glucose (mg/dL)	101.1	102.1	1.2	0.7		
Glucose (mg/dL)	86.2	68.6	0.6	0.0001	0.0001	0.0001
Daily weight gain, kg	0.39	0.17	0.01	0.0001	0.0001	0.0001
Insulin, uIU/mL	182.6	24.3	8.3	0.0001	0.007	0.14
NEFA, µEq/L	156.8	281.1	16.9	0.0001	0.009	0.15

As expected, the hyperglycemic lambs raised on the HC diet had consistently higher concentrations of plasma insulin than lambs grown on the LC diet, with an overall mean of 182.6 vs. 24.3 µIU/mL, respectively (*P* < 0.0001; Fig. 1b; Table 2). Remarkably, within 4-months of the dietary treatment, the HC group had a significantly higher fasting insulin levels (49.1 vs. 20.5 µIU/mL, *P* < 0.0002), despite similar fasting glucose levels (Fig. 1b, Table 3). In other words, the HC group were systemically more resistant to insulin, as indicated quantitatively by their fasting homeostatic model assessment of insulin resistance (HOMA-IR) values of 7.3 compared to 3.1 µIU × mg/[mL]^2^ in the LC group (Fig. 1c; *P* < 0.0008).

**Table 3:** Fasting measurements in the high- and low-calorie treatments.

Parameter	High calorie	Low calorie	SEM	*P-*value (t-test)
Body weight, kg	72.9	45.0	2.9	0.0001
Weight loss, kg	0.8	2.4	0.3	0.006
Glucose, mg/dL	58.9	59.5	0.9	0.8
BHBA, mmol/L	0.313	0.275	0.011	0.09
NEFA, µEq/L	1,010.5	492.9	61.3	0.0001
Insulin, uIU/mL	49.1	20.5	3.9	0.0002

**Figure 2:**
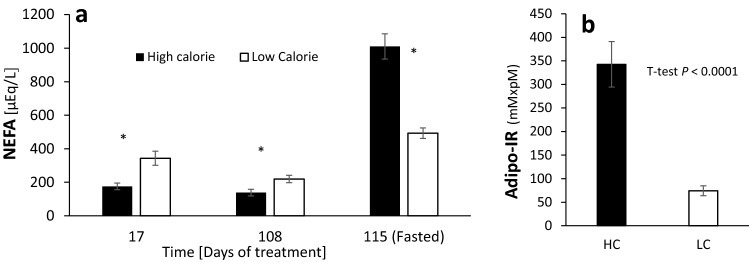
Non-esterified fatty acid (NEFA) and adipose insulin resistance in response to the high- and low-calorie treatments. (**a**) Plasma NEFA concentrations. Repeated measures ANOVA shows an effect of treatment (*P* < 0.0001) and time (*P* < 0.009). SEM = 16.9. Fasting levels were not included in the model and analysed independently by a student’s t-test and the significance level was Bonferroni-Holm corrected. *P < 0.05. (**b**) Adipose tissue insulin resistance as measured by ADIPO-IR. Student’s t-test detects an effect of treatment (*P* < 0.0001) SEM = 34. Due to unequal variances between treatments, the statistical analysis was performed on the log-transformed values.

Intriguingly, despite the higher fasting insulin levels in the HC group (Fig. 1b), their fasting plasma NEFA levels, known to be tightly regulated by insulin, were significantly higher than in the LC group (1,010 vs. 492 µEq/L, *P* < 0.0001, Fig. 2a), indicating adipose tissue insulin resistance. The respective adipose insulin resistance (ADIPO-IR)^18^ index values were 342.6 vs. 74.4 mmol/L × pmol/L; *P* < 0.0001 (Fig. 2b).

### The HC diet induced adiposity, dyslipidemia and enhanced expression of inflammatory cytokines

**Figure 3:**
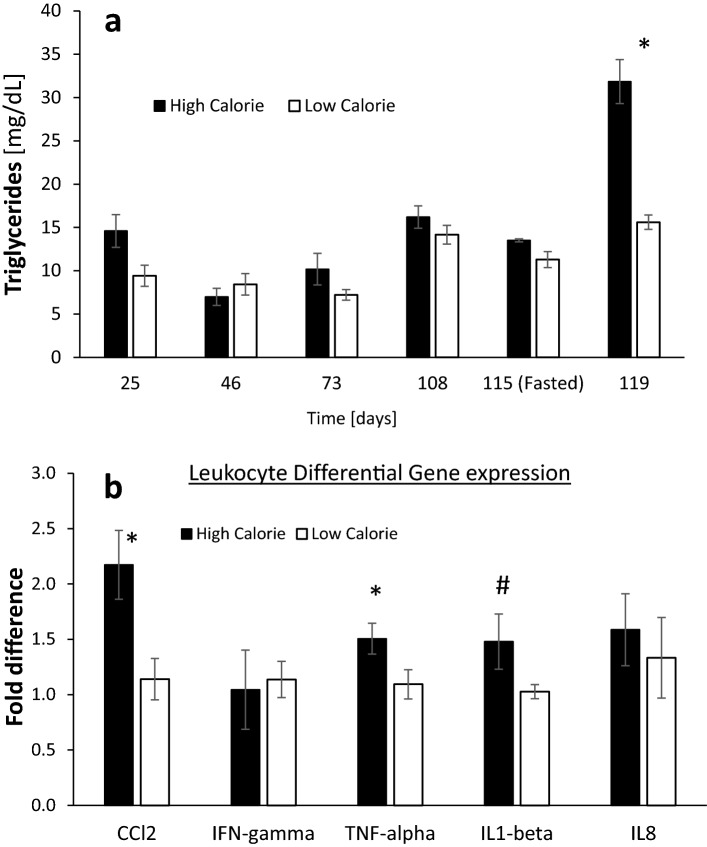
Triglycerides and inflammatory markers in response to the high- and low-calorie dietary treatments. (**a**) Plasma triglycerides concentrations. Repeated measures ANOVA shows an effect of treatment (*P* < 0.0013), time (*P* < 0.0001), treatment by time interaction (*P* < 0.01). SEM = 0.716. Due to unequal variances between treatments, the statistical analysis was performed on the log-transformed values. Data for fasting levels were not included in the ANOVA, and analyzed independently by a Student’s t-test at a *P < 0.05 significance level and Bonferroni-Holm correction. (**b**) Differential gene expression analysis in circulating leukocytes. Student’s t-tests *CCL2 P < 0.01, *TNF-alpha P < 0.04, ^#^*IL1B P* < 0.098.

As expected, lambs raised on the HC diet gained more weight than lambs on the LC diet (average of 392 vs. 165 g/day; *P* < 0.0001), respectively reaching mean final body weights of 73 vs. 45 kg (*P* < 0.0001) (Table 4). The respective final BCS of 3.7 vs. 2.5 (*P* < 0.0001) indicate that the HC lambs developed significant adiposity^19^, as was also reflected in their BMI and GI indexes^20^ (Table 4). Consistent with their enhanced adiposity, after 4 months of treatment, the HC lambs exhibited increased plasma TG concentration (Fig. 3a), with twice the mean final concentration in the LC lambs (31.8 vs. 15.6 mg/dL; *P* < 0.05).

**Table 4:** Physical body parameters in the high- and low-calorie treatments.

	High calorie	Low calorie	SEM	*P-*value (t-test)
Initial BW (kg)	28.3	26.4	0.9	0.28
Final BW (kg)	72.9	45.0	2.9	0.0001
Liver weight (g)	1,365	625	77	0.0001
Hepatic index	1.86	1.39	0.001	0.0001
BCS	3.68	2.53	0.12	0.0001
Body length (m)	1.04	0.86	0.02	0.0001
Wither’s height (m)	0.77	0.72	0.003	0.003
Heart girth (cm)	102.8	92.7	1.6	0.001
BMI (W/BL^2^)	66.8	60.2	1.1	0.002
GI (W/BL^1.5^)	68.2	56.0	1.5	0.0001

SEM: standard error of the mean; BW: body weight; Hepatic Index: (liver weight)*100/(body weight); BCS: body condition score; BMI: Body Mass Index; W: weight; BL: body length; GI: G Index.

Since overnutrition, hyperglycemia, insulin resistance and obesity are associated with systemic low-grade chronic inflammation^21–24^, we investigated the expression levels of several common inflammatory cytokines in circulating leukocytes. The hyperglycemic sheep fed the HC diet showed increased expression levels of the pro-inflammatory markers *CCL2* (*P* < 0.01) and *TNF-alpha* (*P* < 0.04), and a tendency (*P* < 0.098) for higher expression of *IL1B* (Fig. 3b).

### The HC diet induced significant hepatic steatosis and hepatomegaly

**Figure 4:**
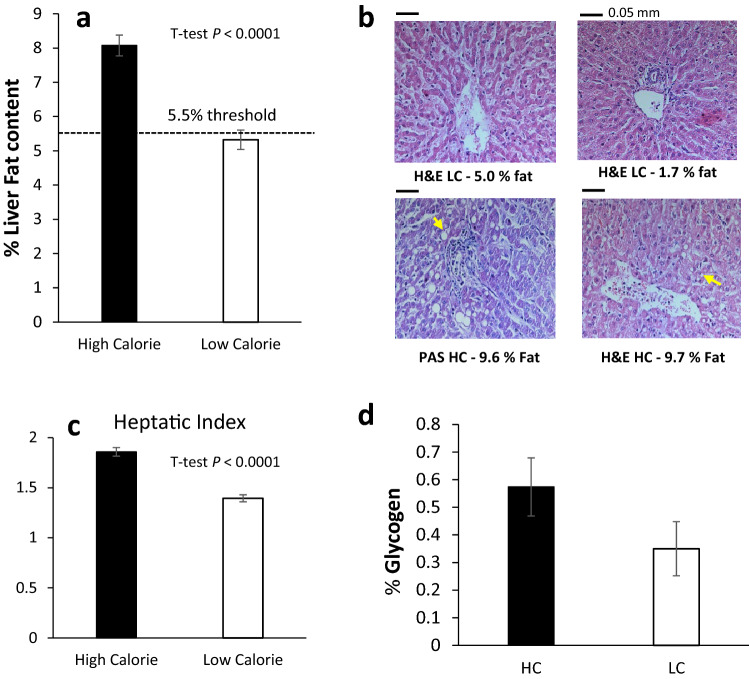
Effect of the high- and low-calorie dietary treatments on the liver. (**a**) Hepatic-fat content, as derived from weight of total fat vs. the wet-liver weight. The HC treatment induced substantial hepatic steatosis, significantly higher than observed for the LC livers (Student t-test, *P* < 0.0001). (**b**) Representative liver tissue sections from the HC and LC groups analyzed by histopathology with Haematoxylin and Eosin (H&E) or Periodic Acid-Schiff (PAS) staining. The HC hepatocytes (bottom) showed macrovesicular and steatosis around both the portal (left) and central (right) veins. The top LC liver slices showed no lipid droplets formation. The images were taken at a ×200 magnification. (**c**) Hepatic index expressed (liver weight)*100/(body weight). Student’s t-test detects an effect of treatment (*P* < 0.0001). (**d**) Percentage of liver glycogen. The values in the HC group trended higher than in the LC group, though not significantly different (student's t-test, *P* < 0.1308; SEM = 0.0735).

Remarkably, the HC group developed substantial hepatic steatosis with an average liver fat content of 8.1 vs. 5.3% in the LC group (*P* < 0.0001; Fig. 4a). By histopathology, the HC group had significantly more extensive microvesicular and macrovesicular hepatocyte steatosis than the LC group (Fig. 4b); with a steatotic score of 2.1 vs. 0.4, respectively (*P* < 0.0002). Occasional lobular inflammation and mild ballooning were also observed among the HC lambs (Supplementary Figure 4), but no significant fibrosis. Intriguingly, lambs in the HC group developed not only larger livers (mean weight of 1.36 vs. 0.62 kg; *P* < 0.0001; Table 4), but also a higher hepatic index (liver weight normalized to body weight; Fig. 4c) with values of 1.9 vs. 1.4 (*P* < 0.0005), respectively, indicative of hepatomegaly.

As may be expected due to their higher energy abundance, the HC group tended to have higher hepatic glycogen content than the LC group (mean of 0.57 vs. 0.35%; Fig. 4d), but the difference was not statistically different (*P* < 0.1308).

### Hyperglycemia was the strongest predictor of hepatic steatosis

A stepwise regression analysis with the liver-fat content as the response variable and the measured physical (body weight, liver weight, hepatic index) and metabolic parameters (average blood glucose, insulin, TG, NEFA, fasting TG, HOMA-IR and ADIPO-IR) as the explanatory variables ("Methods") yielded only two significant effects; average glucose (*P* < 0.0004) and HOMA-IR (*P* < 0.035).

The Pearson correlation analysis of the hepatic steatosis (Table 5) was also the highest with average glucose (r = 0.77; *P* < 0.0001), and as expected, the steatosis clustered well with the dietary treatment (Supplementary Figure 3). The next strongly associated metabolic factors were HOMA-IR (r = 0.68; *P* < 0.0001), mean plasma insulin (r = 0.66; *P* < 0.0001) and triglycerides (r = 0.65; *P* < 0.0001), and ADIPO-IR (r = 0.65; *P* < 0.0001). Hepatic steatosis was poorly associated with fasting plasma-concentrations of glucose (r = 0.26; *P* < 0.163) and with fasting TG (r = 0.24; *P* < 0.198). Surprisingly, although hepatic steatosis was associated with the final fasting plasma NEFA (r = 0.62; *P* < 0.0002), it was negatively correlated with the overall fed plasma NEFA (r = − 0.44, *P* < 0.016). Collectively, these analyses suggest that the observed hepatic steatosis reflects a cumulative effect of the prolonged treatment that is most strongly associated with the average fed-blood glucose, rather than a reflection of the NEFA influx.

**Table 5:** Pearson correlations between liver-fat content and metabolic factors measured in sheep.

Parameter	Pearson correlation (r)	P-value
Fed glucose	0.7735	0.0001
Fasting glucose	0.2614	0.16
Fed insulin	0.6578	0.0001
Fasting insulin	0.6690	0.0001
HOMA IR	0.6775	0.0001
Liver weight	0.7476	0.0001
Liver index	0.6627	0.0001
Fed plasma TG	0.6497	0.0001
Fasting plasma TG	0.24	0.198
Fed plasma NEFA	− 0.4368	0.016
Fasting plasma NEFA	0.6212	0.0002
ADIPO-IR	0.6535	0.0001

## Discussion

### Blood glucose levels reflect the carbohydrates abundance in sheep

The efficient microbial digestion of soluble carbohydrates in the rumen leaves virtually no free dietary glucose for direct intestinal absorption^25^. As such, blood glucose in ruminants originates mainly from endogenous gluconeogenesis based on glucose precursors, primarily propionic acid, derived from the microbial fermentation of their feedstuffs^25^. As the carbohydrates composition of feeds dictate the nature of the fermentation products and their rate of formation^26^, they ultimately dictate the energetic status of the animal. Pelleted concentrate-based feeds are rich in starch and soluble carbohydrates, which metabolize efficiently into propionate in the rumen and provide high levels of metabolizable energy and substrate influx for hepatic gluconeogenesis. At the other extreme, straw and hay-based feeds, which are rich in fibrous structural carbohydrates, ferment less efficiently and provide less metabolizable energy and glucose precursors. Here, we employed two dietary compositions, substantially different in their metabolizable energy, to obtain two groups of sheep considerably differing in their glycemic indexes, as a means to investigate the relationships between high carbohydrate-based energy and hepatic steatosis.

Indeed, the HC concentrate-based ration led to an average consumption of 6.3 Mcal/day of metabolizable energy; yielding an average blood glucose level of up to 100 mg/dL. In sharp contrast, the LC hay-based ration led to an average consumption of 3.5 Mcal/day of metabolizable energy, and average blood glucose of up to 70 mg/dL. Therefore, the blood glucose concentrations in growing lambs fed ad libitum reflected the consumed metabolizable energy, the animals’ carbohydrate-abundance, and their energetic status remarkably well.

### Prolong exposure to excess carbohydrates energy drives systemic metabolic derangements, primarily promoting insulin resistance

The continued ad libitum consumption of the HC diet sustained a sufficient substrate influx for gluconeogenesis to induce steady hyperglycemia throughout the 4 months of the experimental duration. Along with the hyperglycemia, the over-nourished lambs had also developed hyperinsulinemia, excess body fat, dyslipidemia, insulin resistance and increased expression of proinflammatory markers. Humans presenting with these metabolic abnormalities would probably be diagnosed with metabolic syndrome^27^. Of notice, the HC lambs may not be classified as diabetic, at least not during the course of the experimental period, since their final fasting glucose levels were relatively low and statistically non-different from those of the LC lambs (Fig. 1a).

### Excessive carbohydrates levels induce hepatic steatosis

**Figure 5:**
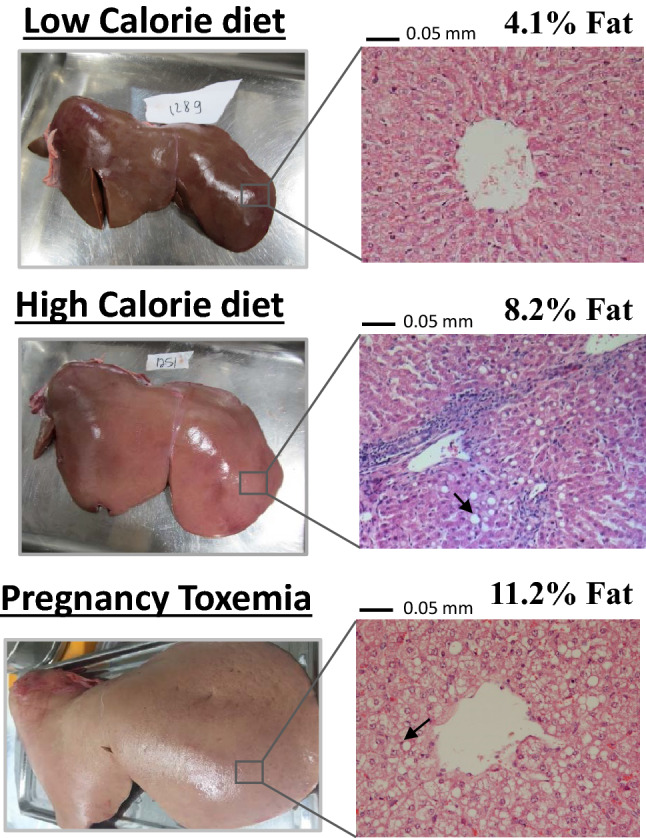
Representative pictures of livers and corresponding histopathological analysis. Liver sections were stained by H&E and imaged at ×200 magnification. The arrows exemplify macrovesicular steatosis where hepatocytes nuclei are pushed aside by large lipid droplets.

The large quantity of liver tissue obtained from the sheep enabled non-subjective fat-content determination at outstanding precision. Whereas lambs in the LC diet developed lean livers (averaged at 5.3% fat), lambs on the HC diet developed steatotic livers ranging from 6.2 to 10.8% (averaged at 8.1% fat). As a pathological reference, one of the fattiest livers documented at the Volcani experimental flock of sheep had a fat content of 11.2%. That liver (Fig. 5) was obtained from a pregnant ewe who died from pregnancy toxemia, the pathogenesis of which has long been attributed to fatty infiltration of the liver due to negative energy balance^28^.

The current study, therefore, demonstrates that ruminants can develop hepatic steatosis, not only due to negative but also due to positive energy balance based on excess carbohydrate energy. Moreover, since both the LC and the HC diets contained very little fat (under 3%), the results suggest that ectopic hepatic-fat accumulation is independent of an exogenous source of fat. Interestingly, this is in contrast to what was found in swine that although develop metabolic syndrome like features on a high-fructose diet, but required substantial dietary fat to develop hepatic steatosis^29^.

Since feed intake is directly linked to lambs growth rates^30^ (Supplementary Figure 2), the HC ration is commonly provided to growing lambs at intensive husbandries. Nevertheless, the clinical consequences of the resulting excessive carbohydrate energy have not been prudently investigated, clearly not concerning the liver. Whether consumption of the HC diet for longer durations may induce advanced liver damage such as fibrosis and or cirrhosis, or whether it potentiates the development of pregnancy fatty liver disease in female lambs grown on it remains to be investigated.

### Initiation of hepatic steatosis may be more dependent on de novo lipogenesis than on adipose lipolysis and likely mediated by hyperinsulinemia

It is commonly believed, both in ruminants and humans, that endogenous fat released from adipose lipolysis, yielding an increased flux of circulating NEFA into the liver, is a major source for hepatic TG accumulation. However, the fed plasma NEFA levels were higher in the LC vs. the HC lambs (Fig. 2a) and thus negatively correlated with hepatic steatosis (Tables 2 and 5). Instead, hyperglycemia was the most steatogenic biochemical parameter, suggesting that hepatic DNL may have directly contributed to the fat accumulated in the liver, and highlights carbohydrates excess as an independent risk factor.

The excess glucose levels can provide substrate flux to feed hepatic DNL (as a carbon source for both FA and glycerol synthesis), but can also signal for upregulation of genes in the FA biosynthetic pathway, either directly via the carbohydrate response element binding protein (chREBP)^31^, or indirectly via insulin-mediated activation of sterol regulatory element binding protein 1C (SREBPc1)^32^. The higher correlation of hepatic steatosis with the hyperglycemia than with the hyperinsulinemia may indicate that the carbohydrate abundance affected steatosis both directly and indirectly via insulin signalling.

Noteworthy, by upregulation of the FA biosynthesis, glucose could potentially contribute to FA synthesis also from the major carbon sources resulting from the microbial ruminal fermentation, i.e., acetate, propionate and butyrate. Although normally acetate is mostly utilized by extrahepatic tissues^33^, propionate is almost exclusively utilized for hepatic gluconeogenesis^34^ and butyrate is largely metabolized to ketone bodies by the rumen^35,36^, it is plausible that, in the extreme hyperglycemic and hyprinsulinemic conditions induced by the HC diet, hepatic DNL was partly based on carbon sources from these metabolites.

### Indifferent glycogen levels may indicate hepatic insulin resistance

The anabolic actions of insulin promote the synthesis of all the major classes of macromolecules, including proteins and the energy storage classes of lipids and glycogen. Accordingly, the hepatic glycogen levels serve as a principal readout for the liver activity of insulin^37^. Since glycogen synthesis requires both the activation of the enzyme glycogen synthase by insulin and the availability of the substrate (glucose 6-phosphate), the combination of hyperinsulinemia and hyperglycemia maximizes glycogen content^38^. Therefore, it can be expected that the HC lambs, which were both hyperglycemic and hyperinsulinemic (Fig. 1), would present with significantly higher levels of hepatic glycogen. The observation that only a moderate increase in glycogen content was observed in the HC vs. the LC lambs (Fig. 4d), may thus suggest that their livers became less sensitive to insulin, which is indeed recognized to be associated with decreased glycogen synthesis^39^.

### Sheep as a large-animal model for fatty liver and metabolic disorder

Although none recapitulates the entire spectrum of the pathophysiology of metabolic steatosis triggered by overnutrition, animal models clearly provide powerful means to investigate disease pathogenesis, to discover disease-modulating targets, as well as to evaluate potential therapeutics in order to increase the probability of success in clinical trials. Since many preclinical results obtained in current rodent models of NAFLD were not translated to humans very successfully, there is significant merit for the development of additional robust models, preferably with better translational potential as large animals of similar body size to human often convey^40^.

This work reveals the potential for using sheep as a highly-controlled large-animal model for FL research, exemplifying the development of hepatic steatosis and key metabolic comorbidities due to excess carbohydrates-based energy, in the absence of an exogenous source of fat. As humans, sheep present with the willingness to overeat, therefore this model reflects the strong environmental component of metabolic and liver disease triggered by overnutrition. Moreover, the development of hepatic steatosis in growing lambs exposed to high carbohydrate-based energy, as shown here, represents the potential for fatty liver development at an early age, which is of potential value to model diet-induced metabolic disorders in children.

The use of sheep as experimental models for obesity^41^ and diabetes^42^, was partly due to practical advantages offered by large animals in physiological studies. Apart from having similar body and liver weights, these include a long life span and a considerably larger quantity of biological material that respectively allow for studying long-term effects and for frequent sampling of the same subject for a broad spectrum of biochemical and molecular applications to investigate tissues- and systemic-related pathologies.

Future molecular-level characterizations and intervention studies to modulate hepatic steatosis will help substantiate the applicability of this model system to the physiological and clinical settings in humans.

### Conclusions

The complexity of the carbohydrates in the diet of ruminants determines the level of glucose precursors and metabolizable energy that can be available to the animal. In growing lambs, it is well reflected in their blood glucose levels. Thus, by feeding lambs with rations of varying consumed metabolizable energy, we obtained two populations of significantly distinct glycemic indexes. The overnourished lambs were consistently hyperglycemic and hyperinsulinemic, and in the course of 4 months, developed adiposity, dyslipidemia, insulin resistance, and also substantial hepatic steatosis and hepatomegaly. Hyperglycemia, representing the high-carbohydrates abundance, was the strongest predictor of hepatic steatosis. Surprisingly, steatosis was negatively correlated with circulating NEFA, suggesting that hepatic DNL may play a more significant role than adipose lipolysis in the initiation of steatosis. The systemic metabolic and liver abnormalities induced by the HC diet in sheep are similar to those observed in humans with metabolic syndrome and liver steatosis; therefore, this large-animal model may be of value to NAFLD research and therapy development.

## Methods

### Animals and experimental design

All procedures involving animals in this study were approved by the Volcani Center Animal Care Committee (permit # 764/18 IL). All methods were carried out in accordance with the relevant guidelines and regulations of this committee. The animal experiments were conducted at the Volcani experimental farm, Rishon LeZion, Israel. The experiment was initiated in December and ended in April, corresponding to the end of winter and the beginning of spring in Israel. The animals were maintained in open-shed pens of a concrete surface, adequately ventilated and illuminated and protected from direct sunlight and rain. After weaning, at an average age of 45 days, lambs were group-fed ad libitum grain concentrate (Table 1) supplemented with oat hay to approximately 8% of the ration until the initiation of the experiment. Thirty-one weaned male lambs of the Afec-Assaf breed^43^ (2.2 ± 0.03 months old, 27 ± 0.85 kg in body weight) were randomly assigned to two treatment groups housed in two adjacent pens. There were seven pairs of siblings, all assigned to different treatments to minimize confounding effects. In practice, in the event siblings were randomly assigned to the same group, one lamb of the pair was swapped with another lamb of similar body weight from the other group. All animals were given a 10-day acclimatization period, during which they were group-fed a concentrate-based diet at ~ 4.8MCal average consumed metabolizable energy. The treatments were either (i) high-calorie (HC) ration (n = 15) based on ad libitum group-feeding concentrate pellets supplemented with 8% oat hay, or (ii) lower-calorie (LC) ration (n = 16) based on ad libitum group-feeding oat hay supplemented with 14% corn and 13% soy to ensure steady growth (Table 1). An equivalent amount of minerals premix was present in both rations. Group left-over feeds were weighed weekly to determine the average intake. The HC lambs had a mean daily intake of 6.3 ± 0.18 MCal metabolizable energy, whereas the LC lambs had a mean daily intake of 3.5 ± 0.21 MCal metabolizable energy, averaged over the last 80 days of the 4-months experimental duration (Supplementary Figure 1).

Blood glucose concentrations were determined weekly using the FreeStyle Optium glucometer (Abbot Diabetes Care Ltd., Oxfordshire, UK)^44^. Body weights were measured weekly. Plasma was isolated monthly. Briefly, 5 mL blood samples were collected via venipuncture of the jugular vein into heparinized vacutainers and immediately placed on ice (Vacutainer; Becton Dickinson and Co., Franklin Lakes, NJ), then centrifuged at 2000×*g* for 15 min at 4 °C. The supernatant (plasma) was collected and immediately stored at − 20 °C until further analysis.

On day 114 of the experiment, all feeds were withdrawn from both treatments for 24 h with free access to water to measure fasting parameters. Before fasting, the animal’s body condition score (BCS) on a scale of 1 to 5 whereby 1 represents very thin and 5 very fat^45^, body length, wither’s height and heart girth were determined. Body weights were measured before and after fasting to determined the fasting weight loss. Fasting blood glucose and BHBA concentrations were measured using the FreeStyle Optium glucometer. Fasting plasma was isolated and stored at − 20 °C until further analysis.

After 121 days in study, the animals were slaughtered and their livers were harvested. About 150 g of the left lobe of the liver was stored in a zip-lock bag at − 20 °C until further biochemical analysis.

### Liver fat content analysis

By histopathology, NAFLD is defined as the presence of ≥ 5% steatotic hepatocytes in the absence of secondary causes such as significant alcohol consumption or use of steatogenic medications^46^. The less subjective biochemical threshold refers to having a total fat weight higher than 5.5% of the wet liver weight, which corresponds to approximately 15% histological steatosis^47,48^. We determined both but based all of our analysis on the more sensitive hepatic fat content quantification by weight. Percent hepatic-fat contents (w/w) were determined as previously described^49,50^ with a slight modification. Duplicates of 1 g of frozen liver samples were homogenized in 25 mL of 2:1 chloroform:methanol solution using a rotor homogenizer. The homogenate was then sonicated (VCX 750, Sonics and Materials Inc., Newtown, CT, USA) for 5 min, 5 s on 5 s off, at an amplitude of 30. Sonicated samples were agitated for 24 h at room temperature and then centrifuged at 3000×*g* for 10 min. The lipids-containing supernatant was collected and washed with 4 mL of 0.9% NaCl to remove polar lipids, then centrifuged at 2500×*g* for 10 min. The resulting upper phase was discarded, and the residual interface was further rinsed twice with 4 mL of 1:1 methanol:water solution. The lower chloroform phase containing the fat (mainly TG and cholesterol esters) was then evaporated under vacuum in a rotary evaporator. The fat was finally oven-dried at 45 °C for 2.5 h to remove residual moisture. The liver fat content was determined as the percentage of the wet liver weight. The average values of the duplicates were used for further statistical analysis.

### Histological analysis

Slices (~ 1 cm × 2 cm × 0.2 cm ) of fresh liver samples immediately after slaughter were fixed in formalin (4% formaldehyde, 13 gr/L sodium phosphate dibasic and 8 gr/L of sodium phosphate monobasic) for at least 60 h before histological analysis. The formalin-soaked samples were dehydrated in a series of ethanol and xylene solutions followed by paraffin embedding. Five µm liver sections sliced using the Microm HM355S microtome (Thermo Fisher Scientific, USA), were fixed on glass slides, then rehydrated with ethanol followed by hematoxylin/eosin (H&E) or periodic acid-Schiff (PAS) staining. Stained sections were mounted in a xylene based mounting media (Tissue-Tek Glas mounting Media, Sakura, Netherlands). Histological images were captured using a digital camera mounted on a Nikon Eclipse 600 microscope (Nikon, Japan). Steatosis was assessed and scored blindly to animal identification by an experienced histopathologist following the NAS scoring system^51^ applied to steatosis only. Assessment was performed using H&E stained sections by counting hepatocytes showing macrovesicular or microvesicular at ×400 magnification in 3 randomly selected fields. Scoring of 0, 1, 2, or 3 was respectively assigned to samples having < 5%, 5–33%, > 33–66% or > 66% steatotic hepatocytes.

### Liver glycogen analysis

Total liver glycogen was isolated as described^52^, with some modifications. Duplicates of 5 g of frozen liver samples were rotor-homogenized in 5 mL of 10% trichloroacetic acid (Sigma-Aldrich, Milwaukee, WI, USA). The homogenate was centrifuged at 3000×*g* for 5 min at 4 °C (as for all subsequent centrifugations), and the supernatant was collected. The above procedure was repeated with the first pellet, and the resulting supernatant was added to the first one. An equal volume of 95% ethanol was added to the total supernatant above, then vortexed and incubated overnight at room temperature for gravitational precipitation of the glycogen, followed by centrifugation. The resulting glycogen pellet was further purified by adding 5 mL of double-distilled H_2_O and 10 mL of 95% ethanol, followed by centrifugation. The pellet was next resuspended in 3 mL of 95% ethanol and centrifuged. Finally, the pellet was resuspended in 3 mL of diethyl ether (Sigma-Aldrich, Milwaukee, WI, USA), centrifuged again and the supernatant was discarded. The purified glycogen pellet was oven-dried at 35 °C for 1 h to remove residual moisture. The glycogen weight was measured, and the glycogen content was determined as the percentage of the wet liver weight. The average values of the duplicates were used for further statistical analysis.

### Plasma biochemical analysis

Plasma NEFA concentrations were determined enzymatically using the NEFA kit (Wako Chemicals, GmbH, Neuss, Germany) according to the manufacture’s instruction. Plasma insulin was analyzed by the radioimmunoassay insulin kit (Coat-A-Count insulin; Diagnostic Products, Los Angeles, CA) according to the manufacturer’s instructions, where 1 IU = 0.0347 mg of human insulin^53^. This kit was previously validated for measurements of bovine, equine and canine^54^, as well as for ovine insulin^55^.

### Leukocyte mRNA quantification

Blood was collected from all sheep on day 119 (one day before slaughter) into EDTA venipuncture tubes and kept on ice for immediate RNA extraction using the leukocyte RNA purification kit (Norgen Biotek Corp., Ontario, Canada) according to the manufacturer’s instructions. DNAase treatment was carried out using the RQ1 RNAase-Free DNase (Promega, Madison, WI, USA). Single-stranded cDNA was synthesized using the Applied Biosystems 2,720 Thermal Cycler (Thermo Fisher Scientific, USA) from 500 ng of total RNA using the Revert Aid RT-PCR Kit (Thermo Fisher Scientific, USA) with the provided random hexamers and oligo(dT) primers, following the manufacturer’s instructions.

Real-time quantitative PCR (RT-qPCR) analysis was carried out on a Rotor gene Q instrument (Qiagen, Hilden, Germany) using the 5 × HOT FIREPol EvaGreen qPCR Supermix (Solis BioDyne, Tartu, Estonia) with *GAPDH* and *YWHAZ* as endogenous control (reference) genes. Gene-specific primers of the target genes (Table S1) were designed to match exon-junctions using the NCBI primer blast server. The RT-qPCR reaction was composed of 4 µL of cDNA, 0.3 µL of each gene-specific primer, 4 µL of 5 × HOT FIREPol EvaGreen qPCR Supermix and completed to a final volume of 20 µL with ultra-pure water (Biological Industries, Kibbutz Beit Haemek, Israel). The reaction program consisted of 12 min incubation at 95 °C and 40 cycles of 95 °C for 15 s, 60 °C for 20 s and 72 °C for 20 s. The geometric mean of the two endogenous reference genes was employed to compute the relative gene expression using the ΔΔC_T_ method^56^ with the mean of the LC treatment as the normalizer.

### Statistical analysis

All animals (N = 31) were treated as independent experimental units. Concentrations of glucose, insulin, NEFA and triglycerides, weight, daily weight gain, in response to the treatments over time were all analyzed as continuous dependent variables with repeated measures ANOVA using the linear mixed models approach in JMP (Version 14.0.0, SAS Institute Inc., Cary, NC, 2018). The model included Treatment (HC vs. LC) as a between-subjects fixed factor, Time (from treatment initiation) as a within-subject fixed factor, Treatment by Time interaction, and Individual Animal as a random factor nested within Treatment. The distributions of the model residuals were visually confirmed for normality. The pretreatment values measured for blood glucose and body weights were non-different between the groups (Tables 2, 4), and therefore were not used as covariate factors in the analysis. Post-hoc pairwise comparisons at particular time points were made by Student’s t-test and corrected for multiple hypotheses testing by the Bonferroni-Holm method^57^. Differences between treatments for variables in which Time was not considered, were also analyzed by Student’s *t-*tests. Unless otherwise stated, data are summarized as means ± standard errors (SE). A significance level of α = 0.05 was employed. Two-tailed *P*-values are shown throughout. The stepwise regression analysis was carried out with *P* < 0.1 as the threshold for the addition and *P* < 0.05 for the subtraction of an effect from the model.

## Supplementary information

Supplementary Information.

## Data Availability

All the data supporting the findings of this study are available within the article and its Supplementary Information.
