# Supplementary material for: Hyperglycemia-stimulating diet induces liver steatosis in sheep
Source: Sci Rep. 2020 Jul 22;10:12189. doi: 10.1038/s41598-020-68909-z (PMC7376193; doi:10.1038/s41598-020-68909-z)
Supplement: Supplementary file 1 — Supplementary Information. [file 41598_2020_68909_MOESM1_ESM.pdf]

Supplementary Information

Hyperglycemia-stimulating diet induces liver steatosis in sheep

Mugagga Kalyesubula[1,2], Ramgopal Mopuri[1], Alexander Rosov[1], Tamir Alon[1,2], Nir Edery[3], Uzi Moallem[1], Hay Dvir[1*]

[1]Institute of Animal Science, Volcani Center - ARO, Rishon LeZion, Israel

[2]Department of Animal Science, the Hebrew University of Jerusalem, Rehovot, Israel.

[3]Pathology Laboratory, Kimron Veterinary Institute, Veterinary Services, Rishon LeZion, Israel

*Correspondence: Hay Dvir, Institute of Animal Science, Volcani Center, ARO, Israel, haydvir@volcani.agri.gov.il

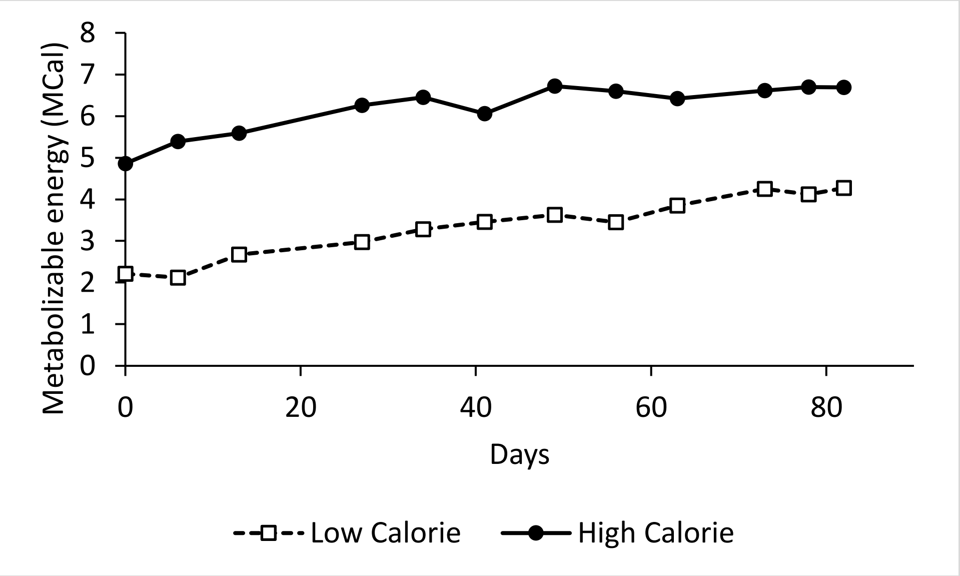

Figure S1: Group caloric intake by the High- and Low-Calorie treatments

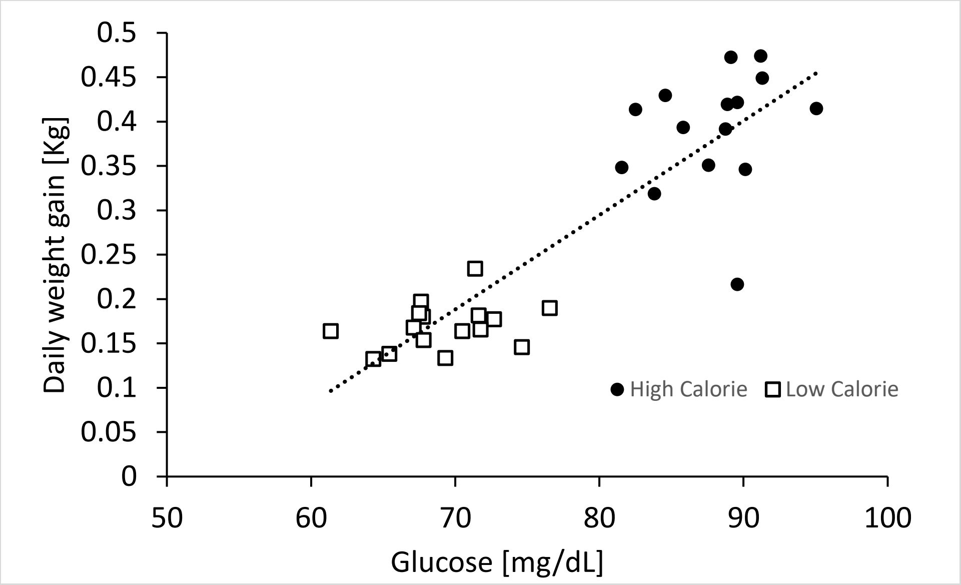

Figure S2. Average daily weight gain per sheep as a function of average blood glucose. The Pearson correlation factor was r = 0.8858 (*P*< 0.0001).

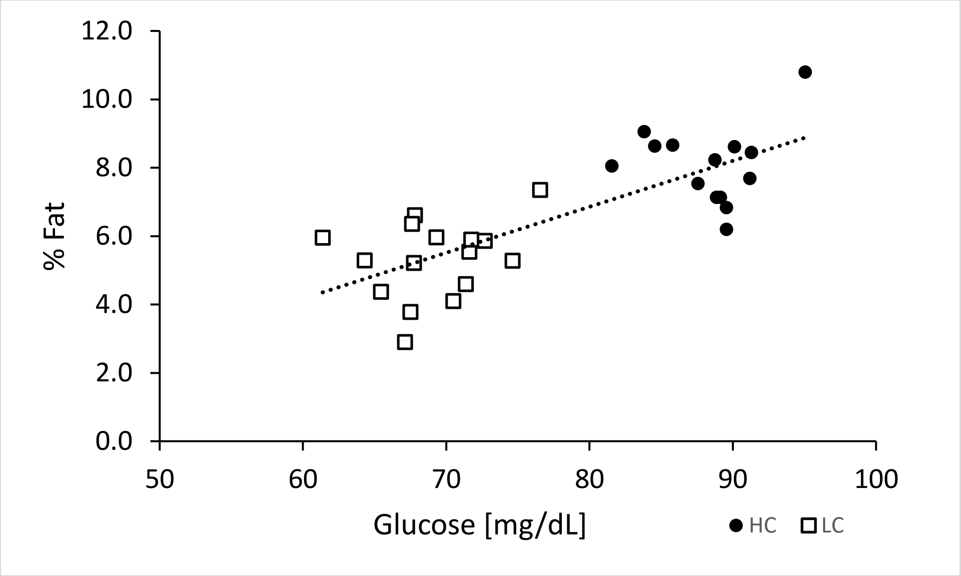

Figure S3. Heptatic fat content as a function blood glucose concentrations. The Pearson correlation was r = 0.7735 (*P*< 0.0001).

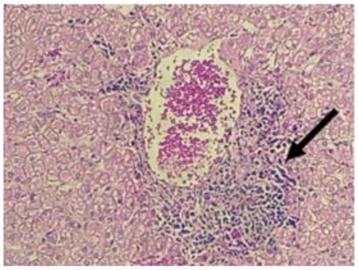

Figure S4. Liver histological analysis in a lamb grown on the high-calorie diet. Hematoxylin and eosin staining of a 5 micrometer liver section imaged at 100x magnification. The arrow marks an area of infiltration of inflammatory cells around a centrolobular vein.

Table S1: Primer sequences employed for real-time quantitative PCR

Gene	GenBank Accession	Primer	Length (bp)
GAPDH	NM_001190390.1	Forward: AAGTTCCACGGCACAGTCAA<br>Reverse: ATGTTGGCAGGATCTCGCTC	92
YWHAZ	XM_027972757.1	Forward: AGACGGAAGGTGCTGAGAAA<br>Reverse: CGTTGGGGATCAAGAACTTT	123
TNFA	NM_001024860.1	Forward: CACGTTGTAGCCAACATCAGC<br>Reverse: GAGGTAAAGCCCGTCAGTGG	129
CCl2 (MCP-1)	XM_004012471.2	Forward: TCGCTCAGCCAGATGCAATTA<br>Reverse: GACACTTGCTGGTGGTGACT	112
IL8	NM_001009401.2	Forward: AAGCTGGCTGTTGCTCTCTTG<br>Reverse: GTGGAAAGGTGTGGAATGTGT	127
IFNG	X52640	Forward: GGAGGACTTCAAAAGGCTGA<br>Reverse: GGTTAGATTTTGGCGACAGG	110
IL1B	NM_001009465.2	Forward: GTGCTGGATAGCCCATGTGT<br>Reverse: CAGAACACCACTTCTCGGCT	74
